# Cardiovascular Magnetic Resonance in Myocarditis

**DOI:** 10.3390/diagnostics12020399

**Published:** 2022-02-03

**Authors:** Christian L. Polte, Emanuele Bobbio, Entela Bollano, Niklas Bergh, Christina Polte, Jakob Himmelman, Kerstin M. Lagerstrand, Sinsia A. Gao

**Affiliations:** 1 Department of Clinical Physiology, Sahlgrenska University Hospital, 41345 Gothenburg, Sweden; christina.linke@vgregion.se (C.P.); sinsia.gao@vgregion.se (S.A.G.); 2 Department of Radiology, Sahlgrenska University Hospital, 41345 Gothenburg, Sweden; 3 Institute of Medicine, The Sahlgrenska Academy at the University of Gothenburg, 41390 Gothenburg, Sweden; emanuele.bobbio@vgregion.se (E.B.); entela.bollano@vgregion.se (E.B.); niklas.bergh@vgregion.se (N.B.); 4 Department of Cardiology, Sahlgrenska University Hospital, 41345 Gothenburg, Sweden; 5 Paediatric Heart Centre Gothenburg, Queen Silvia Children’s Hospital, Sahlgrenska University Hospital, 41650 Gothenburg, Sweden; 6 Department of Medical Physics and Biomedical Engineering, Sahlgrenska University Hospital, 41345 Gothenburg, Sweden; jakob.himmelman@vgregion.se (J.H.); kerstin.lagerstrand@vgregion.se (K.M.L.); 7 Institute of Clinical Sciences, The Sahlgrenska Academy at the University of Gothenburg, 41390 Gothenburg, Sweden

**Keywords:** myocarditis, inflammatory cardiomyopathy, magnetic resonance imaging

## Abstract

Myocarditis is an inflammatory disease of the myocardium, and its diagnosis remains challenging owing to a varying clinical presentation and broad spectrum of underlying aetiologies. In clinical practice, cardiovascular magnetic resonance has become an invaluable non-invasive imaging tool in the evaluation of patients with clinically suspected myocarditis, mainly thanks to its unique multiparametric tissue characterization ability. Although considered as useful, the method also has its limitations. This review aims to provide an up-to-date overview of the strengths and weaknesses of cardiovascular magnetic resonance in the diagnostic work-up of patients with clinically suspected myocarditis in a broad clinical context.

## 1. Introduction

The diagnosis of myocarditis, defined as an inflammatory disease of the myocardium [1], remains challenging owing to its heterogeneity of clinical presentation and broad spectrum of underlying aetiologies [2]. Most frequently, myocarditis is caused by viral infections or other infectious agents, but can also be due to less common causes such as systemic diseases, drugs, or toxins [3,4]. Although mild forms usually resolve spontaneously, myocarditis can also lead to the development of end-stage heart disease owing to a dilated cardiomyopathy [5] or sudden cardiac death [6]. The reference standard for establishing the diagnosis of myocarditis is still endomyocardial biopsy (EMB), although this method faces several limitations, mainly due to its invasive nature, infrequent clinical use, and overall low sensitivity [2,7,8]. In clinical practice, cardiovascular magnetic resonance (CMR) has emerged as a useful non-invasive alternative thanks to its unique multiparametric tissue characterization ability [7]. CMR not only provides diagnostic information concerning the presence of myocardial inflammation [9], but also conveys prognostic information [10,11]. The current position statement regarding myocarditis from the European Society of Cardiology (ESC) Working Group on Myocardial and Pericardial Diseases [2], the ESC Guidelines for acute and chronic heart failure [12], as well as the scientific statement concerning specific dilated cardiomyopathies from the American Heart Association [13] consider CMR a useful method in patients with clinically suspected myocarditis. Despite being useful, CMR faces several limitations, which have to be taken into account in the clinical decision-making process.

Accordingly, the aim of this review is to provide an up-to-date overview of the strengths and weaknesses of CMR in the diagnostic work-up of patients with clinically suspected myocarditis in a broad clinical context.

## 2. Clinically Suspected Myocarditis

### 2.1. Clinical Presentation

Myocarditis can affect patients of all ages, although different underlying aetiologies have their own characteristic age spectrum. In Western countries, most patients suffering from myocarditis are predominantly younger individuals, as most cases are caused by viral infections [2,14].

The spectrum of clinical symptoms is rather wide and unspecific, ranging from mild discomfort due to palpitations, non-specific chest pain, or fatigue to more severe clinical manifestations such as acute coronary syndrome-like presentations, acute (with or without cardiogenic shock) or chronic heart failure, brady- and tachyarrhythmias, as well as conduction abnormalities [15,16]. Infectious prodrome with fever, myalgia, and respiratory or gastrointestinal symptoms can be present in cases of infectious myocarditis, whereas in other cases, symptoms associated with systemic diseases can be of relevance. Owing to the unspecific nature of clinical presentation, many cases of myocarditis may go undetected, are accidentally discovered during autopsy, or are discovered too late when the patient already developed end-stage heart disease [17].

### 2.2. Diagnostic Work-Up

In 2013, the ESC Working Group on Myocardial and Pericardial Diseases proposed new diagnostic criteria to improve the recognition of myocarditis in clinical practice (Table 1) [2]. Hereby, it should be kept in mind that these recommendations were mainly based on viral myocarditis, but can also be extended to other underling aetiologies. Overall, the evaluation of patients with clinically suspected myocarditis is often quite extensive, as multiple differential diagnoses must be considered owing to the frequently unspecific clinical presentation. Furthermore, neither a single clinical nor diagnostic finding can presently confirm or exclude the diagnosis of myocarditis with absolute certainty, which is the reason for using an integrative diagnostic approach [18]. In addition to the clinical history and physical examination, the following diagnostic methods can be of value in the diagnostic work-up of patients with clinically suspected myocarditis:

A 12-lead electrocardiogram is usually pathologic in patients with myocarditis. However, the diagnostic value is rather limited, as the observed electrocardiographic changes are neither specific nor sensitive enough to allow a definite diagnosis [2,7,19].

Biomarkers of myocardial injury (for instance, troponin T or I) and inflammation (for instance, C-reactive protein) can be elevated depending on the severity of inflammation and the timing of the test with respect to the natural course of the disease [20,21]. However, these biomarkers are rather unspecific. In clinical routine, viral testing is generally not recommended because of its unreliability [22].

Echocardiography is an established first-line imaging tool and shows frequently normal or unspecific findings in patients with myocarditis [2,23]. However, echocardiography can help to rule-out other differential diagnoses and is most likely useful for longitudinal follow-up studies, if clinically indicated. A potentially valuable addition is speckle-tracking-derived strain, which might aid in the detection of an acute myocarditis [24,25,26].

A non-invasive coronary computed tomography angiography or invasive coronary angiography is frequently performed in patients with an acute coronary syndrome-like presentation, as well as in other clinical scenarios where it is necessary to rule-out eventual underlying coronary artery disease. Young patients with a classic history consistent with acute myocarditis and no cardiovascular risk factors may be able to obviate a coronary angiography, if immediate access to CMR is available [7].

Fluorine-18 fluorodeoxyglucose positron emission tomography/computed tomography can visualize myocardial inflammation and has become a valuable tool in the complicated diagnostic work-up of patients with clinically suspected cardiac sarcoidosis (CS) as well as their follow-up [27,28,29,30,31]. The method might also be useful in other complicated cases with inconclusive CMR and/or EMB, as for instance in recurrent myocarditis.

Endomyocardial biopsy is currently considered as a reference standard for the diagnosis of myocarditis based on established histological, immunological, and immunohistochemical criteria [1,32]. If performed according to the current indications [33] and in the hands of an experienced operator, EMB has a very low complication rate of <1% [34]. However, the major limitations of EMB are its invasive nature, infrequent clinical use, and overall low sensitivity due to the methods’ sampling limitations and the focal nature of inflammatory cell infiltration. The reported sensitivity is highest in giant cell myocarditis (GCM; approximately 80 to 93% with repeated biventricular sampling), but is much lower in other forms such as lymphocytic myocarditis and CS (estimated to be approximately 20 to 30%) [8,35,36]. These limitations of the current reference standard are not merely a clinical problem, but also an impediment for the introduction of every new diagnostic test, because any difference between the two methods will be held against the new test and not the reference standard.

## 3. CMR Imaging of Myocardial Inflammation

CMR can detect changes caused by myocardial inflammation independent of the underlying aetiology, and has thereby altered the clinical decision-making process of many patients [7,18,37]. The strength of the method lies in its unique multiparametric tissue characterization ability, which, however, relies mainly on parameters exploiting changes in extracellular volume to visualize myocardial inflammation. These visualized aspects of myocardial inflammation are oedema, hyperaemia, capillary leak, necrosis, as well as fibrosis. The extent and intensity of these changes depend on the degree of the underlying myocardial inflammation and the timing of the CMR study in relation to the natural course of the disease, as it progresses from an acute and subacute to a healed or chronic state. This temporal evolution of myocardial inflammation, which often lasts days to weeks before it frequently resolves, limits the optimal sensitivity for diagnostic imaging to only a few weeks from its presentation [38,39]. Thus, it is favourable to perform CMR imaging in the early stage of the disease. Consequently, this may also be the reason CMR performs best in patients with recent onset of angina-like symptoms, but is rather insufficient in patients with heart failure or arrhythmias as their primary symptom [40]. The decreasing inflammatory activity over time, as well as the presence of more diffuse inflammatory processes, which can occur during the transition from the acute to the subacute state and/or in case of underlying autoimmune processes, pose a challenge to classic CMR techniques that require regional signal differences to generate sufficient tissue contrast (for instance, T2-weighted imaging as well as early and late gadolinium enhancement (LGE) imaging) [41]. Therefore, more diffuse or low-grade processes may be left undetected by classic CMR techniques. A potential solution to this problem is the normalization against reference tissue, which enables the calculation of a signal intensity ratio. However, this can lead to false negative results in the case of coexisting disease in the reference tissue, such as, for instance, in skeletal muscle for T2-weighted imaging [42,43]. Nonetheless, in recent years, the method’s overall ability to visualize diffuse myocardial disease has clearly improved thanks to the advances in parametric mapping techniques [44].

### 3.1. CMR Mapping Techniques

**Figure 1:**
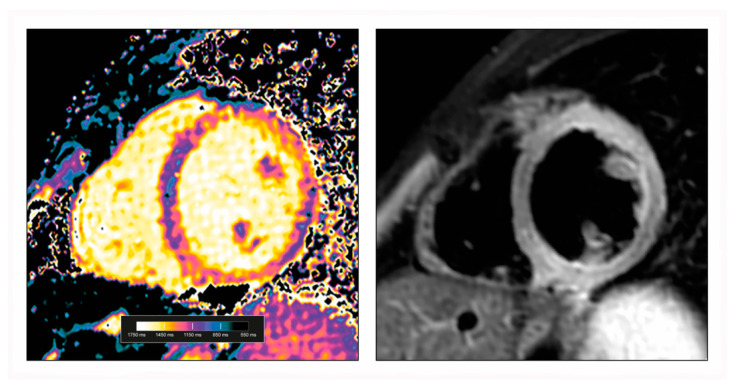
Native T1 map (**left**) in a patient with acute myocarditis showing prolonged T1 relaxation times in the anterolateral and inferoseptal regions of the left ventricle (1320 ± 43 ms (local reference 999 ± 31 ms)). Corresponding T2-weighted black blood short tau inversion recovery sequence (**right**) with clear signs of oedema in the same regions.

Parametric mapping techniques allow not only the spatial visualization, but also the objective quantification of T1 and T2 relaxation times, which are magnetic tissue properties influenced by multiple factors [44]. T1 and T2 relaxation times are displayed as a map (Figure 1), and are calculated on a pixel-by-pixel basis and allow the evaluation of global or regional myocardial T1 and T2 relaxation times. Each deviation from the tissue-specific normal range of these relaxation times (using locally obtained or published values as reference [45,46]) indicates a potential change in tissue composition or disease [44,47,48]. Importantly, local validation against established norms is necessary for these techniques, as the T1 and T2 relaxation times are dependent on the chosen scan method and CMR scanner. T1 relaxation times can be calculated without so-called native T1 mapping, or after the application of gadolinium-based contrast agents. Based on T1 mapping pre- and post-contrast including adjustment for the current haematocrit value, the extracellular volume can be calculated, which may be useful to detect oedema, hyperaemia, capillary leak, and fibrosis [38,47,49,50,51,52,53,54].

Overall, CMR mapping techniques show an excellent sensitivity, specificity, and diagnostic accuracy in patients with clinically suspected myocarditis [55,56]. Current evidence indicates that T2 mapping techniques might be more specific for detecting acute inflammation than T1 mapping, and the method also appears to be more sensitive to detecting oedema in the chronic disease state [38]. This is not surprising, as prolongation of T1 relaxation times is mainly caused by two biological determinates, namely oedema (increase in water due to acute inflammation) and an increase in interstitial space due to fibrosis (later stages of myocardial inflammation or healed state) [44,47].

In contrast, prolongation of T2 relaxations times is solely caused by changes in the tissues water content [44,57,58]. However, T1 and T2 mapping techniques seem to have a complementary diagnostic value, which is the reason both methods are recommended to be part of a comprehensive CMR study in patients with clinically suspected myocarditis [7,44]. Finally, parametric mapping techniques have the potential to enable the diagnosis and follow-up of patients with myocarditis without using gadolinium-based contrast agents.

### 3.2. Myocardial Oedema

A hallmark of myocardial inflammation is the development of oedema, which is mediated by an array of cytokines. An increased tissue water content (oedema) results in the prolongation of both T1 and especially T2 relaxation times (Table 2). These changes can be visualized by several CMR techniques.

Classic T2-weighted imaging, usually using a black-blood short-tau inversion recovery sequence, can visualize myocardial oedema in myocarditis (Figure 1) [49,52,53,54,59,60,61,62,63,64,65,66,67,68,69]. However, the usefulness of the method is often hampered by its low signal-to-noise ratio, susceptibility to arrhythmias and motion, as well as inconsistent image quality [70,71]. According to Lagan et al. [55], the pooled weighted sensitivity, specificity, and diagnostic accuracy of T2-weigthed imaging in myocarditis (based on a qualitative and/or semi-quantitative evaluation) is 62, 76, and 67%, respectively. If image analysis is solely based on the calculation of the T2 signal intensity ratio (≥2.0 is considered as pathologic), the sensitivity and specificity of the method increase to 68 and 91%, respectively [56]. When calculating this ratio, it is advisable to use the serratus anterior muscle as a reference tissue, if accessible [72].

T2 mapping techniques, using gradient or spin-echo sequences with multi-echo readouts, are superior in their ability to detect myocardial oedema compared with classic T2-weighted imaging [38,49,52,53,73,74]. The improved diagnostic performance is attributed to the method’s higher signal-to-noise ratio, fewer motion artefacts (secondary to shorter breath-holds), ability to directly calculate T2 relaxation times, and improved inter- and intra-observer variability as well as diagnostic confidence of T2 mapping. Lagan et al. report a pooled sensitivity, specificity, and diagnostic accuracy for T2 mapping in myocarditis of 72, 87, and 79%, respectively [55]. In a further meta-analysis, Kotanidis et al. report a pooled sensitivity and specificity of 78 and 84%, respectively [56]. Furthermore, the method may have the ability to discriminate active from healed myocarditis [52].

Native T1 mapping, using inversion recovery (for instance, the modified Look-Locker inversion recovery (MOLLI) or shortened modified Look-Locker inversion recovery (ShMOLLI)), saturation recovery (saturation recovery single-shot acquisition (SASHA)), or hybrid sequences, is highly useful in patients with clinically suspected myocarditis [38,49,52,53,54,59,60]. However, native T1 mapping is rather a sensitive marker for myocardial disease in general than for the activity of the disease, as it seems that the method lacks the ability to discriminate acute from chronic disease [52]. Lagan et al. report a pooled sensitivity, specificity, and diagnostic accuracy for T1 mapping in myocarditis of 82, 91, and 86%, respectively [55]. A further meta-analysis by Kotanidis et al. reports a pooled sensitivity and specificity of 89 and 90%, respectively [56].

Finally, one must bear in mind that myocardial oedema can not only occur in cases of myocardial inflammation, but might also be due to other myocardial diseases such as, for instance, advanced decompensated heart failure with venous congestion [75].

### 3.3. Myocardial Hyperaemia and Capillary Leak

Myocardial inflammation also leads characteristically to hyperaemia, an increased vascular permeability, and an expansion of the interstitial space, which results in a prolongation of the T1 relaxation time as well as an increased uptake and distribution volume for gadolinium-based contrast agents (Table 2). On the one hand, the prolongation of the T1 relaxation time can be visualized and quantified by native T1 mapping [53,59]. On the other hand, the increase in contrast uptake can be depicted by T1-weighted spin echo sequences before and early after the application of gadolinium-based contrast agents. This enables the calculation of the early gadolinium enhancement ratio (≥4.0 is considered as pathologic) using skeletal muscle as reference, or the contrast media-induced relative myocardial signal intensity increase (≥45% is considered as pathologic) [18]. Although considered useful in patients with clinically suspected myocarditis [49,53,54,61,63,64,65,68,69,76], it is still unclear whether these methods really visualize hyperaemia or if they just reflect an increase in interstitial space. Lagan et al. report a pooled sensitivity, specificity, and diagnostic accuracy for classic early gadolinium enhancement in myocarditis of 65, 69, and 67%, respectively [55]. In a further meta-analysis Kotanidis et al. report a pooled sensitivity and specificity of 70 and 74%, respectively [56]. Interestingly, the removal of early gadolinium enhancement from the original Lake Louise Criteria (LLC), which were proposed in 2009 to establish diagnostic CMR criteria for diagnosing patients with clinically suspected myocarditis, does not appear to substantially affect the diagnostic performance [7,64].

### 3.4. Myocardial Necrosis and Fibrosis

The occurrence of myocyte injury due to severe myocardial inflammation leads to tissue necrosis and eventually to fibrosis and the development of a remaining scar. This results, among others, in the further increase in the distribution volume of gadolinium-based contrast agents, which can be visualized using inversion-recovery prepared gradient echo sequences following a delay after contrast injection (Table 2). The resulting classic LGE images are of great value in patients with clinically suspected myocarditis [38,49,52,53,54,59,60,61,63,64,65,66,67,68,77,78,79], and show characteristic non-ischemic patterns (a typically patchy appearance with most often subendocardial or mid-wall localization). These LGE patterns are discussed in more detail for each specific myocarditis form in Section 5. LGE is not specific for myocardial inflammation, as it only reflects changes in extracellular space, and cannot reliably differentiate between a more recent or an older episode of myocarditis. Furthermore, the extent of LGE decreases and the signal intensity increases owing to the temporal evolution of myocardial inflammation, as the oedema subsides, and the scar develops [80]. Lagan et al. report a pooled sensitivity, specificity, and diagnostic accuracy for LGE in myocarditis of 63, 85, and 72%, respectively [55]. A further meta-analysis by Kotanidis et al. reports a pooled sensitivity and specificity of 68 and 96%, respectively [56].

### 3.5. Functional and Pericardial Alterations

Myocardial inflammation can also lead to structural or functional alterations of the heart, such as, for instance, a swollen myocardium, regional wall motion abnormalities, ventricular dilatation, or impairment of diastolic/systolic function. Furthermore, myocardial inflammation can be associated with pericardial involvement, or vice versa. This may result in a pericardial effusion or characteristic CMR findings consistent with pericarditis [81]. Both functional and pericardial alterations are considered as supportive diagnostic criteria [7,18].

Finally, a further strength of CMR in the diagnostic work-up of patients with clinically suspected myocarditis is its ability to exclude other potentially underlying differential diagnoses.

## 4. Updated Lake Louise Criteria

The original LLC [18] have been used extensively in both clinical and research settings [38,49,53,54,63,64,65,68]. In a meta-analysis by Lagan et at., the pooled sensitivity, specificity, and diagnostic accuracy for the original LLC are 77, 81, and 79%, respectively [55]. A further meta-analysis by Kotanidis et al. reports a pooled sensitivity and specificity of 78 and 88%, respectively [56]. Furthermore, both meta-analyses underline the additional diagnostic potential of parametric mapping techniques as a complement to the classic CMR techniques in the diagnostic work-up of patients with clinically suspected myocarditis.

In 2018, an updated version of the LLC was published [7], which incorporated for the first time CMR mapping techniques into the diagnostic algorithm, as they offer at least theoretically a diagnostic advantage over the original LLC. The updated LLC (Table 3) proposed a “2 out of 2” approach for the diagnosis of myocardial inflammation, which means that one positive T2-based criterion (T2-weigthed imaging or T2 mapping) and one T1-based criterion (T1 mapping, extracellular volume, or LGE) must be fulfilled. Hereby, it should be kept in mind that fulfilling both a positive T2- and T1-based marker increases the specificity of the diagnosis, although the presence of only one positive marker (either T2- or T1-based) makes the diagnosis still likely, albeit with less specificity [7].

## 5. CMR in Different Forms of Myocarditis

The appearance of myocarditis on CMR images is versatile, owing to the broad spectrum of underlying aetiologies (Table 4).

### 5.1. Viral Myocarditis

**Figure 2:**
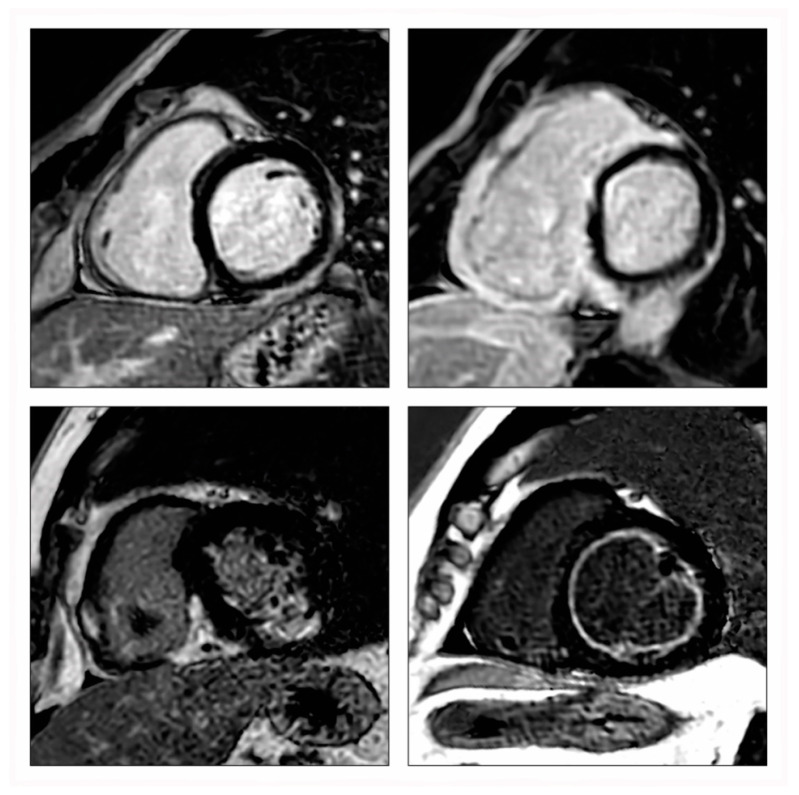
Characteristic late gadolinium enhancement (LGE) patterns in viral myocarditis (**upper left**, inferolateral subepicardial LGE), giant cell myocarditis (**upper right**, complex LGE involving both ventricles including the right ventricular insertion points), cardiac sarcoidosis (**lower left**, complex LGE involving both ventricles including the inferior right ventricular insertion point), and eosinophilic myocarditis (**lower right**, diffuse subendocardial LGE with high signal intensity).

Viral myocarditis is the most common form in Western countries and has been extensively studied by CMR. It shows a LGE pattern that involves the subepicardial and/or mid-wall layers of the myocardium, predominately in the basal to mid-lateral and inferolateral wall segments of the left ventricle (Figure 2). Frequently, all CMR makers of myocardial inflammation resolve within 5 weeks after the initial presentation [39], but sometimes, sequelae in the form of a scar can remain. CMR has even shown its usefulness in cases of chronic viral myocarditis [82], in which LGE is an important marker that can be found in up to 70% of patients with biopsy-proven chronic inflammation [83].

### 5.2. COVID-19 and Post-Vaccination Associated Myocarditis

Coronavirus disease 2019 (COVID-19) is caused by the severe acute respiratory syndrome coronavirus 2 (SARS-CoV-2) and leads to various cardiac manifestations, including signs of myocardial injury [84,85,86]. Numerous cases have been described with clinically suspected myocarditis [87,88,89]. However, only in a few cases histological evidence of lymphocytic infiltration or the presence of the SARS-CoV-2 genome could be found [90,91]. Based on the current available evidence, viral myocarditis seems to be rare in patients with COVID-19, and the underlying pathomechanism is still somewhat unclear.

Myocarditis following mRNA COVID-19 vaccination is a rare complication in predominately male adolescent and young adults, usually of mild nature [92,93]. In these cases, CMR usually shows classic signs of myocarditis as in viral myocarditis [94]. Still, the long-term outcome of vaccine-related myocarditis remains to be clarified.

### 5.3. Giant Cell Myocarditis

GCM is a rare, often rapidly progressive disease, which usually affects middle-aged adults and carries a high mortality if untreated [95]. The diagnosis of GCM remains challenging and relies mainly on EMB to establish the final diagnosis. CMR often shows signs of extensive inflammation with widespread LGE involving all myocardial layers as well as both ventricles including the right ventricular insertion points (Figure 2) [96,97,98,99]. The pattern can look like severe forms of CS [100], which is not surprising as both diseases show a certain clinical and histopathologic overlap. However, the CMR appearance of GCM has so far not been systematically studied.

### 5.4. Cardiac Sarcoidosis

Sarcoidosis is a chronic, multisystem, granulomatous disease of unknown aetiology, which leads in about 5% of the cases to cardiac involvement with various clinical symptoms [101]. CMR is an established and valuable diagnostic tool in the complicated diagnostic work-up of patients with clinically suspected CS as well as their follow-up [27,102]. The CMR appearance of CS is highly variable depending on the stage of disease and shows varying LGE that can involve all myocardial layers as well as both ventricles including the right ventricular insertion points (Figure 2) [103,104]. Furthermore, it has been shown that CMR has a complementary diagnostic value in combination with Fluorine-18 fluorodeoxyglucose positron emission tomography/computed tomography [28], and adds valuable information concerning prognosis in this challenging patient group [105,106].

### 5.5. Eosinophilic Myocarditis

Eosinophilic myocarditis is characterized by eosinophilic infiltration of the myocardium [107]. It is a rare disease that can be caused by hypersensitivity, allergic reactions, infections, malignancies, vasculitis, and hypereosinophilic syndromes [108]. CMR tends to visualize a diffuse subendocardial LGE pattern with high signal intensity [108,109], which stands in clear contrast to all other forms of myocarditis (Figure 2).

### 5.6. Myocarditis in Systemic Immune-Mediated Diseases

Myocarditis can occur as a complication of systemic immune-mediated diseases such as systemic lupus erythematosus, rheumatoid arthritis, and systemic sclerosis. In these cases, CMR can, with the help of classic as well as novel mapping techniques, detect both subclinical and clinically manifest cardiac involvement [110,111,112,113].

### 5.7. Immune Checkpoint Inhibitor-Induced Myocarditis

Immune checkpoint inhibitors are gaining increasing importance in modern cancer treatment. Despite their better safety profile in comparison with chemotherapy, immune-related adverse events can occur [114]. Immune checkpoint inhibitor-induced myocarditis is such a complication and CMR has shown promising results in this patient group [115,116]. However, further studies are needed to systematically characterize the CMR appearance of immune checkpoint inhibitor-induced myocarditis.

### 5.8. Myocarditis in Children and Adolescence

Myocarditis in the paediatric population is rare and remains, as in adults, a difficult diagnosis owing to its heterogenous clinical presentation and broad spectrum of underlying aetiologies. Overall, infectious myocarditis, particularly due to viral infections, is the most frequent form. Using the same diagnostic criteria as in adults, the clinical usefulness of CMR, including parametric mapping techniques, has been demonstrated in the diagnostic work-up of paediatric patients with clinically suspected myocarditis [117,118,119,120,121]. However, the overall evidence for CMR imaging in this patient group is still scarce.

## 6. Future Directions

There is an urgent need for more research in the field of myocarditis. Large outcome-based studies are needed to substantiate the usefulness of parametric mapping techniques and the updated LLC in the diagnosis of myocarditis. New imaging markers based on advanced image analysis techniques, such as texture analysis [122,123] and myocardial strain analysis using feature tracking [124], may help to improve the diagnostic and prognostic ability of CMR. Furthermore, hybrid imaging techniques like PET/MR with the potential to also visualize myocardial inflammation on a molecular level might further improve the diagnostic capabilities. One such promising method for molecular inflammation imaging is somatostatin receptor imaging [125,126,127]. Finally, randomized controlled trials are needed to improve the treatment of patients with inflammatory heart disease.

## 7. Conclusions

CMR is an established and highly valuable clinical tool in the diagnostic work-up of patients with clinically suspected myocarditis. The method’s strength lies in its multiparametric tissue characterization ability and the established diagnostic criteria for the detection of myocardial inflammation. Although widely used in clinical practice, the method also faces several limitations, which can hopefully be resolved in the near future.

## Figures and Tables

**Table 1:** Diagnostic criteria for clinically suspected myocarditis.

Clinical presentation *
Acute chest pain, pericarditic, or pseudo-ischemic New onset (days up to 3 months) or worsening of dyspnoea at rest or exercise, and/or fatigue, with or without left and/or right heart failure signs Subacute/chronic (>3 months) or worsening of dyspnoea at rest or exercise, and/or fatigue, with or without left and/or right heart failure signs Palpitation, and/or unexplained arrhythmia symptoms and/or syncope, and/or aborted sudden cardiac death Unexplained cardiogenic shock
Diagnostic criteria
ECG/Holter/Stress test features New findings (any of the following): atrioventricular block I–III, bundle branch block, ST-segment and T-wave alterations, reduced R wave height, abnormal Q waves, low voltage, sinus arrest, frequent premature beats, supraventricular tachycardia, ventricular tachycardia or fibrillation, and asystole
2. Biomarkers of myocardial injury Elevated troponin I or T
3. Cardiac imaging Echocardiography/Angiography ○ New regional wall motion or global systolic or diastolic function abnormality, with or without ventricular dilatation, with or without increased wall thickness, with or without pericardial effusion, with or without endocavitary thrombi Cardiovascular magnetic resonance ○ New regional wall motion or global systolic or diastolic function abnormality, with or without ventricular dilatation, with or without increased wall thickness, with or without pericardial effusion, with or without endocavitary thrombi ○ Oedema, and/or hyperaemia, and/or late gadolinium enhancement of classic myocarditic pattern

Clinically suspected myocarditis if ≥1 clinical presentation and ≥1 diagnostic criteria from different categories, in the absence of (1) angiographically detectable coronary artery disease (coronary stenosis ≥50%); (2) known pre-existing cardiovascular disease or extra-cardiac causes that explain the symptoms (for instance, valve disease, congenital heart disease, hyperthyroidism, and so on). The suspicion increases with higher numbers of fulfilled criteria. * If the patient is asymptomatic, ≥2 diagnostic criteria should be met. Adapted and modified with permission from Caforio et al. [2].

**Table 2:** CMR features in myocarditis according to disease stage and pathologic findings.

	Oedema	Hyperaemia/Capillary Leak	Necrosis	Fibrosis (Focal/Diffuse)
**Acute** (active)	T2↑ (*T2 SIR/T2 map*) T1↑ (*native T1/ECV*)	T1↑ (*native T1/ECV*) EGE - or +	LGE - or +	-
**Chronic**	T2 - or↑ (*T2 SIR/T2 map*) T1 - or↑ (*native T1/ECV)*	T1 - or↑ (*native T1/ECV*) EGE - or +	LGE - or +	LGE - or + T1 - or↑ (*native T1/ECV*)
**Healed**	-	-	-	LGE - or + T1 - or↑ (*native T1/ECV*)

Native T1 and ECV reflect not only acute inflammation with oedema, but also the later stages with focal or diffuse fibrosis. ECV, extracellular volume; EGE, early gadolinium enhancement; LGE, late gadolinium enhancement; T2 SIR, T2 signal intensity ratio; ↑, increased; -, absent/normal; +, present.

**Table 3:** Updated Lake Louise Criteria.

Main criteria (“2 out of 2”)
**T2-based imaging** ○ Regional * high T2 signal intensity *or* ○ Global T2 signal intensity ratio ≥2.0 in T2-weighted images *or* ○ Regional or global increase of myocardial T2 relaxation times ** **T1-based imaging** ○ Regional or global increase of native myocardial T1 relaxation times or extracellular volume *** *or* ○ Areas with high signal intensity in a non-ischemic distribution pattern in late gadolinium enhancement images
Supportive criteria
**Pericardial inflammation** ○ Pericardial effusion in cine images *or* ○ High signal intensity of the pericardium on late gadolinium enhancement images and/or T1 mapping and/or T2 mapping **Left ventricular dysfunction** ○ Systolic left ventricular wall motion abnormality in cine images

* Regional refers to an area of at least 10 continuous pixels. ** Published or local normal values. *** T1 mapping is highly sensitive to detecting both acute and chronic forms of increased free water content within the myocardium, and thus is considered as an alternative criterion to early gadolinium enhancement. If paired with late gadolinium enhancement to diagnose myocarditis, the areas of T1 abnormality should be beyond that detected by late gadolinium enhancement. Adapted and modified with permission from Ferreira et al. [7].

**Table 4:** Characteristic features of several forms of myocarditis.

	Viral Myocarditis	Cardiac Sarcoidosis	Giant Cell Myocarditis	Eosinophilic Myocarditis
**Demographics**	Mostly young adults, both genders	Mostly middle-aged, both genders	Mostly middle-aged, both genders	Mostly adults <40 years, both genders
**Most common clinical presentation**	Acute coronary syndrome-like with eventual infectious prodrome	Ventricular arrhythmia, heart block, worsening heart failure- Often associated with extra-cardiac sarcoidosis	Ventricular arrhythmia, heart block, worsening heart failure	Acute coronary syndrome-like with fever and dyspnoea
**Clinical course**	Entire spectrum from asymptomatic to fulminant course	Entire spectrum from asymptomatic to fulminant course	Usually fulminant course	Usually acute
**Characteristic LGE pattern**	Subepicardial and/or mid-wall LGE, predominantly basal to mid-lateral and inferolateral wall segments	Varying, usually complex * LGE involving both ventricles including right ventricular insertion points	Often extensive, complex * LGE involving both ventricles including right ventricular insertion points	Diffuse subendocardial LGE with high signal intensity

* Can involve all myocardial layers. LGE, late gadolinium enhancement.
